# Ecdysone Induced Gene Expression Is Associated with Acetylation of Histone H3 Lysine 23 in *Drosophila melanogaster*

**DOI:** 10.1371/journal.pone.0040565

**Published:** 2012-07-10

**Authors:** László Bodai, Nóra Zsindely, Renáta Gáspár, Ildikó Kristó, Orbán Komonyi, Imre Miklós Boros

**Affiliations:** 1 Department of Biochemistry and Molecular Biology, University of Szeged, Szeged, Hungary; 2 Chromatin Research Group, Hungarian Academy of Sciences, University of Szeged, Szeged, Hungary; 3 Institut de Génétique et de Biologie Moléculaire et Cellulaire, CNRS UMR 7104 - Inserm U 964, Illkirch, France; 4 Institute of Biochemistry, Biological Research Centre, Hungarian Academy of Sciences, Szeged, Hungary; Ludwig-Maximilians-Universität München, Germany

## Abstract

Posttranslational modification of histones regulates transcription but the exact role that acetylation of specific lysine residues plays in biological processes *in vivo* is still not clearly understood. To assess the contribution of different histone modifications to transcriptional activation *in vivo*, we determined the acetylation patterns on the ecdysone induced *Eip74EF* and *Eip75B* genes in *Drosophila melanogaster* larvae by chromatin immunoprecipitation. We found that acetylation of histone H3 lysine 23 is localized to promoters and correlates with endogenous ecdysone induced gene activation. In contrast, acetylation of lysines 8, 12 and 16 of histone H4 and lysine 9 of histone H3 showed minor differences in their distribution on the regulatory and transcribed regions tested, and had limited or no correlation with ecdysone induced transcriptional activity. We found that dCBP, which is encoded by the *nejire* gene, acetylates H3 lysine 23 *in vivo*, and silencing of *nejire* leads to reduced expression of the *Eip74EF* and *Eip75B* genes. Our results suggest that acetylation of specific lysine residues of histones contribute specifically to the dynamic regulation of transcription. Furthermore, along with previous studies identify CBP dependent H3 lysine 23 acetylation as an evolutionarily conserved chromatin modification involved in steroid induced gene activation.

## Introduction

The hereditary material of eukaryotes can be found in a complex structure called chromatin, which beside DNA also contains protein and RNA molecules. The basic building blocks of chromatin are the nucleosomes that consist of a protein core, containing two of each histone proteins H2A, H2B, H3 and H4, wrapped around twice with 146 bp of DNA [1]. The organization of nucleosomes and posttranslational modifications (PTMs) of histone proteins play a pivotal role in the regulation of DNA dependent nuclear processes by modulating the accessibility of the chromatin template [2]. Histones can be covalently modified by a variety of chemical appendages ranging from small functional groups to whole proteins, like ubiquitin or SUMO [3]. These modifications may alter the interaction of histones with DNA, neighboring nucleosomes and chromatin binding proteins. The observation that a high number of histone PTMs affect the unstructured N-terminal histone tails not involved in the formation of the nucleosome core particle, and the identification of protein domains responsible for the binding of modified residues of histones favors the idea that PTMs act primarily by providing binding surfaces to chromatin associated proteins i.e. rendering the chromatin more or less accessible to these factors [4]. This notion forms the basis of the histone code hypothesis that proposes that combinations of histone PTMs recruit specific binding factors thereby lead to specific functional outcomes [5].

Acetylation of lysine residues, one of the firstly described histone PTMs [6], affects the lysine rich N-terminal tails of all four core histones [4]. As acetylation neutralizes the positive charge of lysine residues first it was proposed that it loosens chromatin by weakening the association of the negatively charged DNA with the protein core of the nucleosome. Later characterization of the acetyl-lysine binding bromodomain [7], which can be found in several chromatin binding proteins, proved that this PTM can also exert its effect by recruiting chromatin binding proteins. The spatial and temporal pattern of histone acetylation is established by the opposing action of two enzyme groups, the histone acetyltransferases (HATs) and the histone deacetylases (HDACs), both consisting of several conserved protein families [8], [9]. Histone acetylation is dynamically regulated, with a usual half-life of 2–3 minutes that rarely exceeds 30–40 minutes [10]. The high turn-over rate strongly suggests that instead of influencing epigenetic memory acetylation participates in the regulation of dynamic processes on the chromatin template.

Although a large body of knowledge had been accumulated about the characteristics and biological functions of histone acetylation, information about its pattern and role during dynamic transcriptional changes *in vivo* is scarce. Therefore, we designed experiments to reveal histone acetylation patterns associated with gene activation in *Drosophila in vivo*. Loss of several HAT genes in flies (for example *gcn5* [11], *enoki mushroom* [12] or *chameau* [13]) causes lethality during the larva - pupa transition or during metamorphosis, suggesting that histone acetylation might play an essential role in the 20-hydroxyecdysone (20E) induced transcription response. 20E is the steroid molting hormone responsible for the timing of transitions between developmental stages [14]. In its target cells 20E binds to and activates its receptor, a heterodimer of the Ecdysone Receptor and Ultraspiracle proteins [15], then the activated hormone-receptor complex acts as a transcription factor to induce the expression of its target genes [16]. The primary targets of the activated receptor, the so called early genes, are themselves transcriptional factors that activate the expression of late (effector) genes [17]. Thus, release of the hormone leads to a well regulated gene activation cascade. We investigated histone acetylation patterns on different functional regions of the *Eip74EF* [18] and *Eip75B* [19] ecdysone induced early genes during their activation in late third instar larvae. The acetylation of lysine 23 of histone H3 was associated with the promoters of activated ecdysone induced genes, while other residues (H3K9, H4K8, H4K12 and H4K16) showed little or no change in their level of acetylation in response to 20E. We found, that H3K23 acetylation is catalyzed by dCBP, the product of the *nejire* gene, *in vivo* and that normal dCBP function is required for the proper activation of the *Eip74EF* and *Eip75B* genes.

## Results

### *Eip74EF* and *Eip75B* Promoters are Induced in Late L3 Larvae

Mutations of several histone acetyltransferase genes cause lethality in late L3 larvae or in early pupae when pulses of the molting hormone ecdysone trigger metamorphosis by activating the transcription of ecdysone responsive genes. Therefore, we hypothesized that acetylation of nucleosomal histones plays a significant role in the regulation of the ecdysone response and by analyzing the pattern of acetylation on ecdysone induced genes during this period we can gain insight into the role of this PTM in gene regulation *in vivo*.

**Figure 1:**
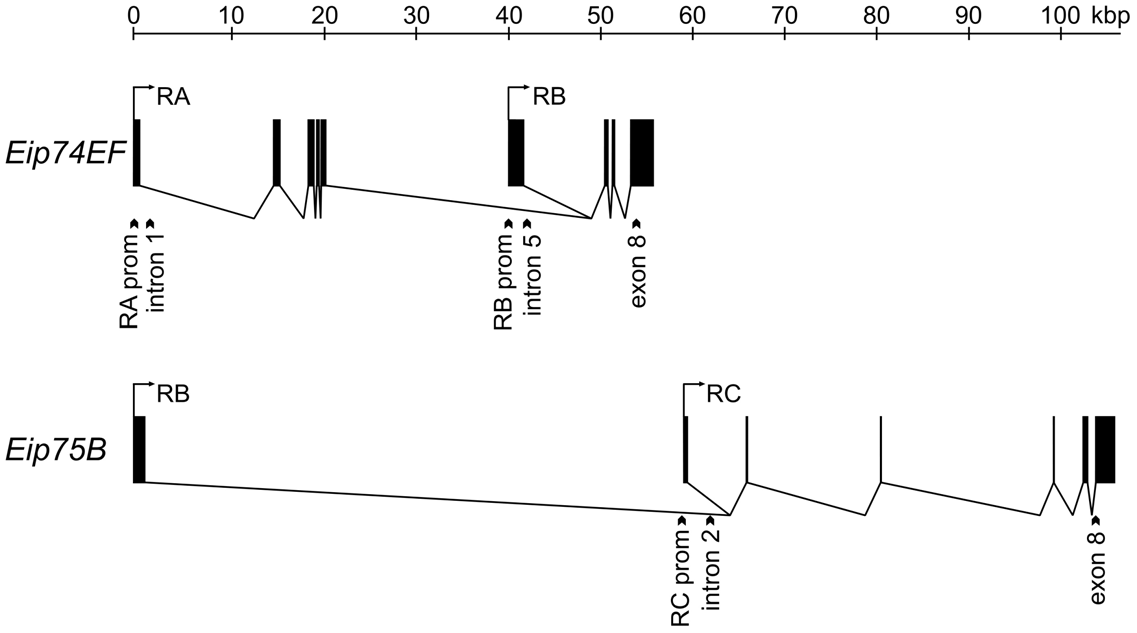
Gene models of the *Eip74EF* and *Eip75B* genes. Characteristic transcript variants of the two genes are shown and labeled based on the *Drosophila* genome annotation by FlyBase [51]. Exons are represented by solid rectangles, promoters are marked by arrows. Arrowtails show the position of amplicons detected in Q-PCR based transcript analysis and ChIP assays. These amplicons fall to the following regions: *Eip74EF-RA* promoter (RA prom), *Eip74EF-RA* 5′ transcribed region (intron 1), *Eip74EF-RB* promoter (RB prom), *Eip74EF-RB* 5′ transcribed region (intron 5), *Eip74EF* 3′ transcribed region (exon 8), *Eip75B-RC* promoter (RC prom), *Eip75B-RC* 5′ transcribed region (intron 2) and *Eip75B-RC* 3′ transcribed region (exon 8). The sequences and chromosomal location of primers are in Table S1.

**Figure 2:**
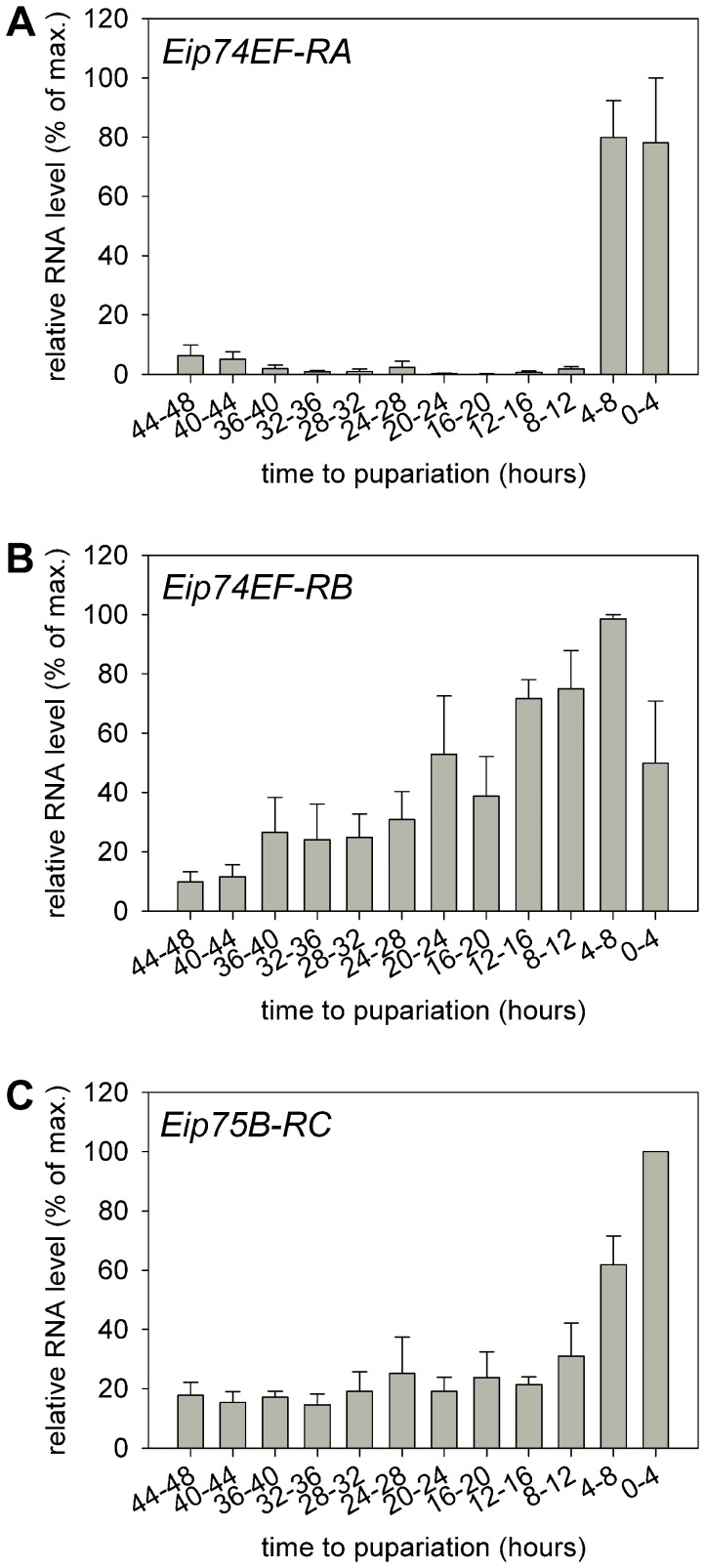
Transcriptional activity of *Eip74EF* and *Eip75B* promoters during the third larval instar. Transcript levels from the *Eip74EF-RA* (A), *Eip74EF-RB* (B) and *Eip75B-RC* (C) promoters in *w^1118^* larvae are shown. The charts show the average of relative RNA levels in percent of the maximum RNA quantity measured with the given primer pairs, the error bars indicate the standard error.

Our aim was to characterize inducible promoters that are activated in late third larval instar (L3) when the level of 20-hydroxyecdysone is elevated. Therefore, we determined the transcriptional activity profile of the *Eip74EF-RA*, *Eip74EF-RB* and *Eip75B-RC* promoters during the L3 stage in four hours resolution. The *Eip74EF* and *Eip75B* ecdysone induced genes are complex transcription units that express several transcript variants from alternative promoters (Fig. 1). Promoter activity was determined by Q-PCR using primers specific for intronic regions 1–2 kb downstream of the selected promoters that amplify only cDNAs that are derived from nascent RNAs but not those from accumulated mRNAs. The *Eip74EF-RA* promoter is silent during the first 40 hours of the L3 stage and is strongly activated 8 hours before pupariation during the wandering L3 (w-L3) stage (Fig. 2A). The *Eip74EF-RB* promoter is active throughout the L3 stage. Its activity is moderately increased during the second day of L3, reaches its highest level 4–8 hours before pupariation then it is downregulated (Fig. 2B). The *Eip75B-RC* promoter shows low transcriptional activity during the first 40 hours of the L3 stage; then it is activated in wandering larvae and reaches its peak level in the last four hours before pupariation (Fig. 2C). Thus, we found that all of the three selected promoters are activated during the late third larval ecdysone pulse and they show a somewhat different transcriptional profile.

### Histone Modification Patterns on the *Eip74EF* and *Eip75B* Genes

**Figure 3:**
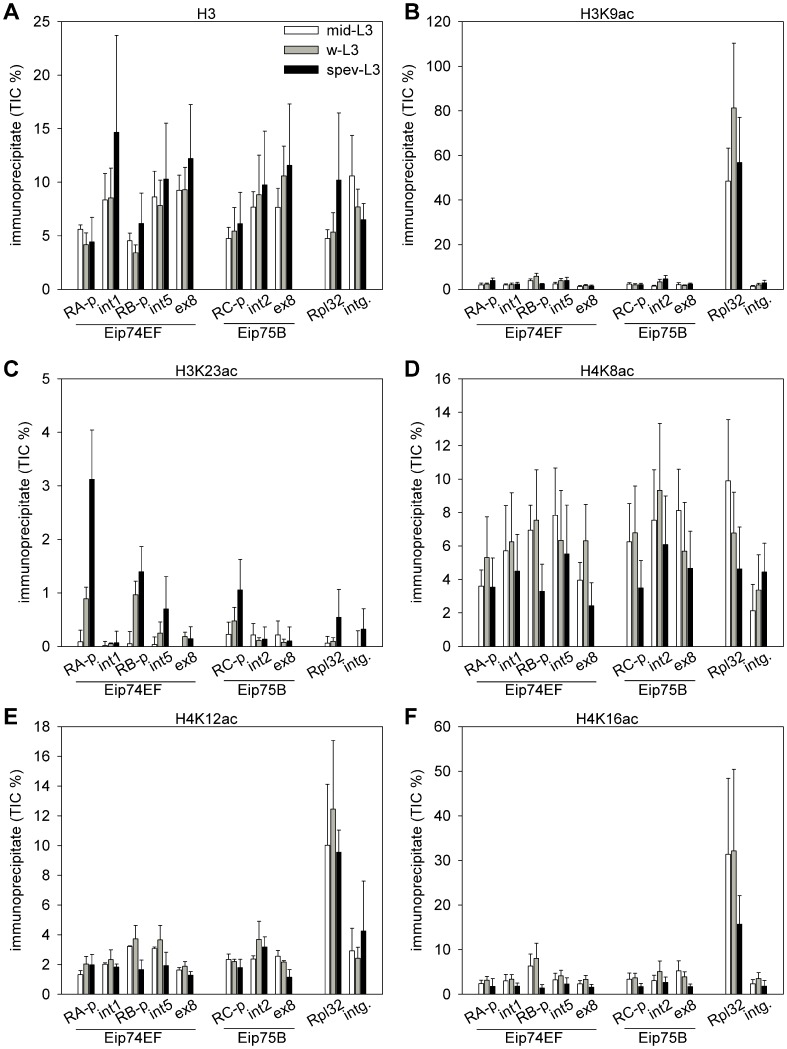
Specific histone acetylations show different spatial and temporal patterns on ecdysone induced genes. Chromatin samples from middle-stage L3 larvae (mid-L3, empty bars), from wandering L3 larvae (w-L3, grey bars) or from late L3 larvae prior pupariation (spev-L3, black bars) were immunoprecipitated with antibodies against (A) histone H3, (B) acetyl-H3K9, (C) acetyl-H3K23, (D) acetyl-H4K8, (E) acetyl-H4K12 or (F) acetyl-H4K16, then quantitated by real-time PCR. In case of histone H3 (A), the amount of precipitated DNA is expressed in the percent of the total input control (TIC). Charts B-F shows the relative quantities of the DNA precipitated by the specific antibodies normalized to the amount of the H3 precipitate, the average ± s.e.m. of three biological replicates are show. (Percentage values do not compare between different antibodies.) The primers detected the following regions: RA-promoter, intron 1, RB-promoter, intron 5 and exon 8 of *Eip74EF*; RC-promoter, intron 2 and exon 8 of *Eip75B*, the promoter of *Rpl32* and a euchromatic intergenic control region.

To determine the pattern of histone modifications during the activation of the *Eip74EF* and *Eip75B* genes, we collected *w^1118^* (wild-type) larvae for chromatin immunoprecipitation (ChIP) analysis at three time points representing different stages of ecdysone response. We collected synchronized larvae in the middle of the L3 stage (mid-L3), which has low ecdysone level; wandering L3 larvae (w-L3) in which the ecdysone level is elevated; and from larvae with everted anterior spiracles (spev-L3), which are immediately before pupariation and has the highest ecdysone level. The occurrence of nucleosomes and acetylated histones on functionally distinct gene regions was determined by ChIP of larval chromatin samples with antibodies raised against histone H3 or against specific acetylated histones, followed by Q-PCR quantitation (Fig. 3). The PCR primers used amplified sequences of promoters, 5′ introns and 3′ exons of the *Eip74EF-RA*, *Eip74-RB* and *Eip75B-RC* transcriptional units (Fig. 1), and also two control regions: one at the promoter of the highly expressed *Rpl32* ribosomal protein gene, and another in an euchromatic intergenic region.

By measuring the levels of histone H3 we found that all regions in question were occupied by nucleosomes at all time points (Fig. 3A). The level of histone H3 did not change significantly at any of the regions tested during gene induction. However, significant differences were detected between the investigated gene regions during the w-L3 and spev-L3 stage that could be mainly contributed to the low nucleosome occupancy of the *Eip74EF-RA* and *-RB* promoters. It is important to note that there was no significant difference in H3 level between the intergenic control region and the transcribed regions of the *Eip74EF* and *Eip75B* genes; or between the four investigated promoters, i.e. the three ecdysone inducible ones and the constitutively highly active *Rpl32* promoter.

In order to identify chromatin marks present at the *Eip74EF* and *Eip75B* genes we tested a panel of antibodies recognizing histone H3 or H4 proteins acetylated or methylated at specific lysine residues in a pilot ChIP experiment, and found that acetylated forms of the H3K9 (lysine 9 of histone H3), H3K23, H4K8, H4K12 and H4K16 residues could be reliably detected. Thus, we determined the pattern of these PTMs during the late L3 stage ecdysone response.

The H3K9 residue was highly acetylated on the *Rpl32* control gene, while all other regions exhibited more than ten times lower level of acetylation, indicating that this modification is primarily associated with highly active gene expression (Fig. 3B). We did not find significant differences between the *Eip74EF* and *Eip75B* gene regions and the intergenic control region at any time point, nor significant changes in H3K9 acetylation at any region between the three time points. These results suggest that the level of H3K9 acetylation we observed at the *Eip74EF* and *Eip75B* genes is not directly linked to ecdysone induced transcription.

Acetyl-H3K23 could be primarily detected at regulatory regions of the *Eip74EF* and *Eip75B* genes, the level of this modification was significantly higher on the *Eip74EF-RA* and *–RB* promoters in the w-L3 and spev-L3 stages than on other regions (Fig. 3C). Furthermore, the level of H3K23 acetylation significantly increased during the ecdysone response on the *Eip74-RA* and *–RB* promoters. In contrast to the ecdysone induced genes, acetyl-H3K23 levels were low on the *Rpl32* promoter. Thus, the dynamics of H3K23 acetylation showed characteristics that implicate this PTM in ecdysone dependent gene regulation.

Although the level of H4K8 acetylation shows a seemingly even distribution, several gene sequences (*Eip74EF-RB* promoter and intron 5, *Eip75B* intron 1 and exon 8, *Rpl32*) is enriched in this modification compared to the intergenic control region in the mid-L3 sample (Fig. 3D). Furthermore, a statistically significant general decrease of acetyl-H4K8 levels was detected in the spev-L3 samples compared to the mid-L3 or w-L3 samples.

The level of H4K12 and H4K16 acetylation (Fig. 3 E and F, respectively) showed a pattern resembling to H3K9 acetylation i.e. they were enriched on the highly expressed *Rpl32* gene, but showed minor regional or temporal changes on the *Eip74EF* and *Eip75B* genes. However, similarly to H4K8 acetylation, a general decline in acetyl-H4K16 levels was observed in the spev-L3 stage.

### *Drosophila* CBP has H3K23 Specific Acetyltransferase Activity

Among the H3 and H4 specific histone modifications tested acetylation of H3K23 was the only one that showed strong specificity to the promoters of *Eip74EF* and *Eip75B* and correlated with their activation suggesting that this modification is involved in the regulatory steps leading to gene induction. To be able to modulate the level of this PTM we aimed to identify the HAT enzyme specific for it in flies. Previous studies have implicated the acetyltransferases GCN5 in yeast [20], [21] and CBP in mammals [22], [23] in the acetylation of the H3K23 residue, thus, we considered the *Drosophila* orthologs of these factors as candidates.

**Figure 4:**
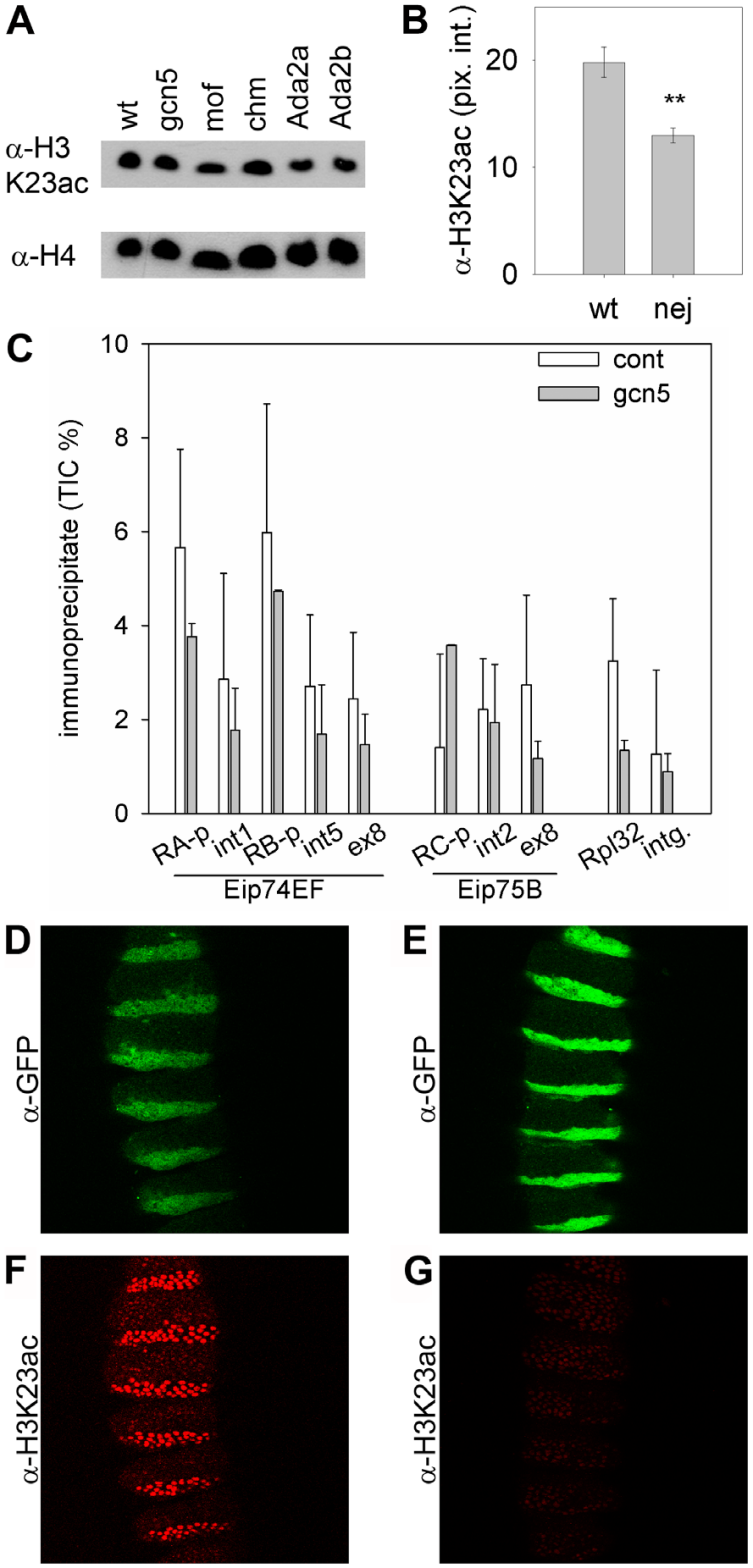
The nej/dCBP protein is responsible for H3K23 specific acetylation in *Drosophila*. (A) The level of acetylated H3K23 is unchanged in L3 larvae homo- or hemizygous for loss of function mutant alleles of the *gcn5*, *mof* or *chm* histone acetyltransferase genes, and also in homozygous *Ada2a* or *Ada2b* mutants specific for the GCN5 containing ATAC and SAGA complexes, respectively. (B) In *nej^3^* mutant embryos the level of acetyl-H3K23 is significantly reduced compared to the wild-type as quantitated by immunostaining of whole mount embryos. Mean pixel intensity ± s.e.m. are shown, P<0.01. (C) ChIP analysis of chromatin samples from *gcn5^E333St^* homozygous null mutant (gcn5) and heterozygous (cont) wandering L3 larvae using acetyl-H3K23 specific antibody detects the presence of the acetyl-H3K23 mark in *gcn5* mutants. In *engrailed-GAL4 UAS-GFP* embryos also carrying either an *UAS-dCBP* (D) or an *UAS-dCBP-FLAD* (E) transgene, the UAS transgenes are expressed in the posterior part of every segment, as visualized by GFP fluorescence. Immunostaining with anti-acetyl-H3K23 specific antibody reveals that the level of acetyl-H3K23 is dramatically increased in embryos overexpressing *UAS-dCBP* (F), while it is unchanged in embryos overexpressing the *UAS-dCBP-FLAD* construct (G), which is mutated in the acetyltransferase domain of CBP.

*Drosophila* GCN5 is the catalytic subunit of the ATAC and SAGA acetyltransferase complexes, which can be inactivated by mutating the complex specific ADA2a or ADA2b subunits, respectively. Null mutations of the *gcn5, Ada2a* or *Ada2b* genes cause lethality at the end of the third larval instar or in early pupae [11], [24], therefore we analyzed the level of acetyl-H3K23 in late L3 larvae in which GCN5 activity supposed to reach critically low levels. We tested samples from homozygous *gcn5^E333St^*, *Ada2a^189^* and *Ada2b^842^* mutants next to homozygous *chm^14^*, *mof^2^* (two H4K16 specific HATs [25], [26]) and *w^1118^* (wild-type) controls by western analysis and found that loss of neither of these factors led to decreased H3K23 acetylation (Fig. 4A).

To rule out the possibility, that although GCN5 is not required for the maintenance of global H3K23 acetylation levels but it is required specifically for the acetylation of the same residue on the *Eip74EF* and *Eip75B* genes, we performed ChIP experiments on chromatin samples derived from *gcn5^E333St^* homozygous and heterozygous (control) wandering L3 larvae (Fig. 4C). We found that acetyl-H3K23 can be detected in *gcn5* mutants, although its level is slightly diminished at several tested regions. As previously we have found, that at this developmental stage *gcn5^E333St^* larvae are protein null [27], and the acetylation of main GCN5targets, such as H3K9 and H3K14 nearly completely vanishes [28], we concluded that GCN5is not, or at least not solely responsible for H3K23 acetylation.

Next we asked whether dCBP/nejire, the single *Drosophila* ortholog of the mammalian CBP and p300 proteins is responsible for the acetylation of the H3K23 residue. As loss of dCBP activity in *nej^3^* null mutants results in embryonic lethality [29], we investigated the consequences of loss of dCBP function in embryos. By comparing the intensity of anti-acetyl-H3K23 immunostaining of whole mount wild-type and hemizygous *nej^3^* embryos we found that the level of acetyl-H3K23 immunoreactivity was significantly lower (P<0.01) in *nej* mutants (Fig. 4B). The residual acetyl-H3K23 immunoreactivity in *nej^3^* mutants can be attributed to maternal effects as it was reported previously that dCBP mRNA and protein is present in *nej* mutant embryos at a reduced level [30], although we cannot exclude the possibility that other HATs are also capable of acetylating this residue.

To determine whether dCBP harbors H3K23 specific acetyltransferase activity, we overexpressed UAS-dCBP or UAS-dCBP-FLAD transgenes, the latter of which is acetyltransferase dead [31], along with a UAS-GFP marker under the control of an engrailed-GAL4 driver in embryos. The engrailed-GAL4 driver activates the expression of UAS transgenes in the posterior half of each segment as is visualized by anti-GFP immunostaining (Fig. 4D and 4E). Anti-acetyl-H3K23 immunostaining reveals similar pattern in embryos carrying UAS-dCBP, i.e. strong anti-acetyl-H3K23 immunoreactivity can be observed in the posterior part of every segment, where dCBP is overexpressed, while only weak staining is detected in the anterior parts of the segments where the UAS-dCBP transgene is silent (Fig. 4F). In contrast, in embryos overexpressing the UAS-dCBP-FLAD transgene increased anti-acetyl-H3K23 immunoreactivity cannot be observed (Fig. 4G). We observed similar staining pattern by detecting the acetylation level of H3K27, a previously characterized substrate [32] of dCBP (Fig. S1). Taken together these findings indicate that dCBP participates in the acetylation of the H3K23 residue *in vivo.*

### dCBP is Required for Larval development and Proper *Eip74EF* and *Eip75B* Transcription

**Figure 5:**
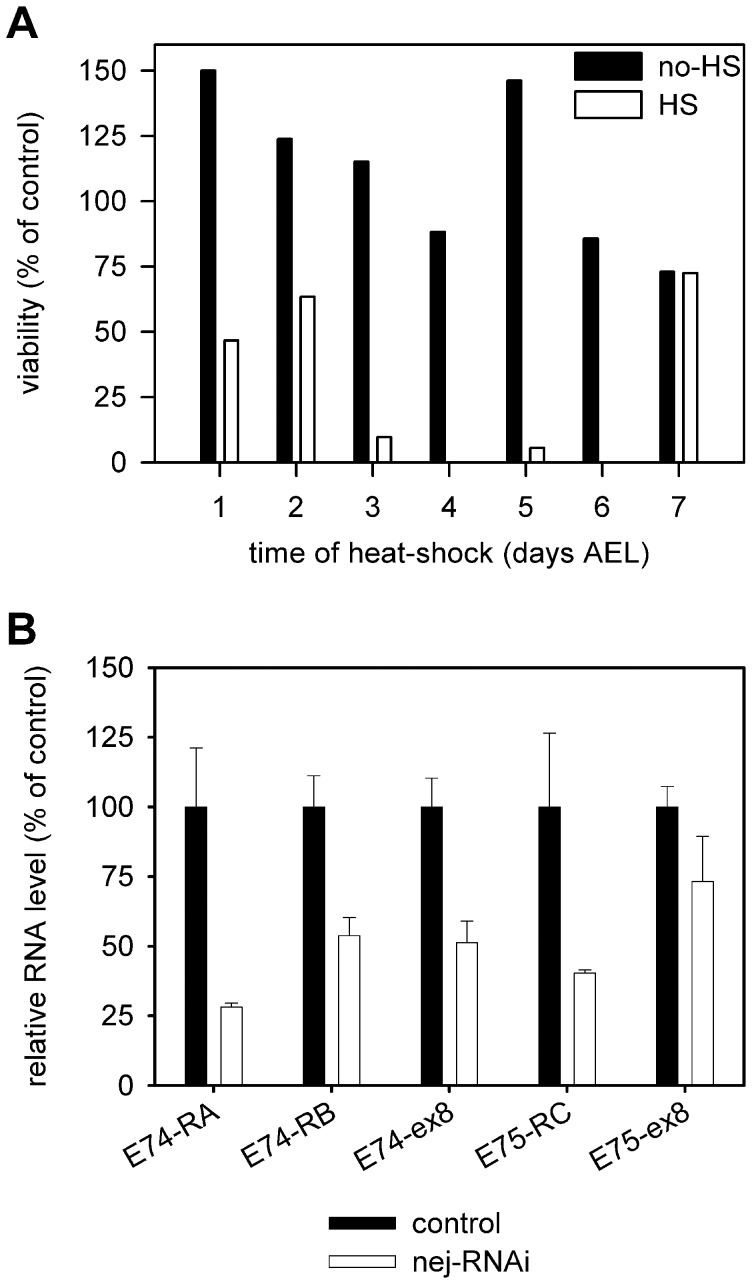
dCBP is required for larval development and ecdysone induced gene expression. (A) Heat-shock activated one-off expression of a *UAS-nejire-RNAi* construct in *hs-GAL4> UAS-nej-RNAi* animals (empty bars) reduces viability compared to non-heat-shocked controls (filled bars). Especially strong effect could be observed if the heat-shock was administered between days 3 and 6 after egg laying. Viability is expressed as the ratio of the number of eclosed *hs-GAL4> UAS-nej-RNAi* flies and *UAS-nej-RNAi* non-expressing control siblings. (B) The level of *Eip74EF* and *Eip75B* specific transcripts is decreased in *nej-RNAi* expressing *hs-GAL4> nej-RNAi* wandering L3 larvae (empty bars) compared to non-expressing control siblings (filled bars). Quantitative PCR analysis was performed with intronic primer pairs specific for the *Eip74EF-RA*, *-RB* or *Eip75B-RC* promoters (E74-RA, E74-RB and E75-RC, respectively), and with primer pairs located in downstream exons detecting mRNA products of the two genes (E74-ex8 and E75-ex8). The chart shows the relative RNA quantities normalized to transcript levels in non-expressing controls ± s.d.

The early lethality of *nejire* mutants hinders the assessment of dCBP functions in larval development, therefore we turned to the use of inducible RNAi lines. To be able to determine whether dCBP is essential in specific developmental stages we generated flies that carried *UAS-nejire-RNAi* constructs along with a heat-shock inducible *hs-GAL4* driver, and activated the expression of the *nejire-RNAi* construct by a single heat-shock on specific days of development from day 1 to day 7 after egg laying. As Figure 5A shows, without induction the viability of *hs-GAL4> UAS-nejire-RNAi^KK105115^* flies was not significantly lower than that of *UAS-nejire-RNAi^KK105115^* control siblings. However, one-off heat-shock seriously compromised the viability of *hs-GAL4> UAS-nejire-RNAi^KK105115^* flies. Especially strong reduction in viability was observed if the heat-shock was between days 3 to 6, i.e. during the L2 or L3 instars or in the prepupal stage, while much weaker or no effect could be seen if the heat-shock was in day 1, 2 or 7 after egg laying, corresponding to the embryonic, L1 larval or pupal stages (Fig. 5A). This suggests that dCBP is required for larval development and the prepupal-pupal transition, but is not essential for pupal development or residual dCBP could be sufficient to promote development at this stage. The resistance of embryos and L1 larvae to the effects of *nejire-RNAi* could be attributed to maternally deposited dCBP.

After we established that dCBP is required during larval development, we sought to determine whether expression of *nejire-RNAi* influences the expression of the *Eip74EF* and *Eip75B* genes. Therefore, we crossed *UAS-nejire-RNAi^KK105115^* females with *hs-GAL4/TM6 Tb* males and compared the expression of the *Eip74EF* and *Eip75B* genes in *nejire-RNAi/hs-GAL4* versus *nejire-RNAi/TM6 Tb* control larvae after heat-shock (Fig. 5B). We observed dramatic reduction in the activity of the *Eip74EF-RA* and *-RB* and the *Eip75B-RC* promoters. The amount of accumulated *Eip74EF* mRNA decreased to half, while a small reduction could be detected in the level of the *Eip75B* mRNA.

## Discussion

### Acetylation of Specific Lysines Mediate Specific Functions

Acetylation of nucleosomal histones is generally thought to be associated with active transcription, although the exact role of specific acetyl-lysines is often not clearly elucidated. The understanding of the functional consequences of histone acetylation is made difficult not only by the variety of molecular interactions (histone - DNA, histone – chromatin binding factor, and intra- and internucleosomal histone - histone) it can alter but also by the often contradictory findings different experimental approaches provide. To resolve these discrepancies a two-step model had been proposed by Anamika *et al.* according to which distinct histone acetylation patterns are established by HATs recruited in a specific manner in the initiation phase of transcription, which then attract additional HATs with different substrate specificities in the latter, maintenance phase [33]. In this study, we found that the acetylation of specific lysine residues showed markedly different patterns during the induction of the *Eip74EF* and *Eip75B* genes, suggesting that they contribute to different biological functions. The patterns of modifications we observed fall into three major categories: first, acetylation of H3K23 that showed strong spatial and temporal specificity; second, acetylation of H3K9, H4K12 and H4K16 that showed moderate changes and low levels on the ecdysone induced genes but was accumulated on the highly expressed *Rpl32* ribosomal protein gene; and last, acetylation of H4K8 that showed moderate differences considering all tested regions and time points. Thus, among these modifications only acetyl-H3K23 is accumulated on regulatory sequences of the *Eip74EF* and *Eip75B* genes and correlates with their transcription, suggesting that this PTM is involved in their ecdysone induced upregulation. All other modifications tested show largely uniform distribution on the two ecdysone induced genes with acetylation levels comparable to those on the intergenic control region, suggesting that PTMs other than acetyl-H3K23 are not directly associated with the ecdysone response. However, we cannot exclude the possibility that moderate alterations in the levels of these modifications are masked in chromatin samples derived from whole larvae used in this study. Although both *Eip74EF* and *Eip75B* are expressed in most if not all larval and imaginal tissues during the late L3 stage [34], [35], the expression of their various transcript variants are not uniform but show differences in timing and tissue distribution [34], [36]. It is important to note, that our results do not fully support the above described two step model, because the increase in the level of the initiating H3K23 acetylation is not followed by increased acetylation of the other analyzed residues on these genes. This pattern falls in line with previous observations describing the relation of H3K23 acetylation to other modifications. In a genome wide study, acetyl-H3K23 was found in chromatin domains that are under Polycomb-mediated repression and in active regions that are poor in the active marks acetyl-H3K9, acetyl-H4K16 and trimethyl-H3K4 [37].

### Conserved Epigenetic Patterns on Steroid Induced Genes

Steroid hormones regulate the development and cellular and physiological functions of vertebrate and invertebrate species and also play an important role in human health and disease. They exert their effects primarily through inducing transcriptional changes with the aid of their receptors, members of the conserved nuclear hormone receptor family, which act as transcription factors. Besides ligand binding, the activity of these receptors is also influenced by posttranslational modifications, interaction with co-factors and the chromatin environment. Protein acetylation is involved in the regulation of steroid hormone action in at least two levels: acetylation of the receptor and acetylation of nucleosomal histones. Homologs of nejire/dCBP participate in both processes. For example, the human p300 protein acetylates the androgen receptor [38] and the estrogen receptor α (ERα) [39] while CBP acetylates the thyroid hormone receptor α [40], the modifications resulting in enhanced ligand dependent transactivating activities and modulated DNA or ligand binding affinities of the receptors. At the chromatin level, p300 is recruited to the promoters of thyroid hormone responsive genes and required for their proper expression and thyroid induced metamorphosis in *Xenopus* tadpoles [41]. Human CBP is recruited to both promoter and enhancer regions of androgen receptor target genes in a ligand dependent manner where it acetylates core histones [42]. CBP is also recruited to estrogen induced gene promoters upon estrogen induction resulting in sequential acetylation of the H3K18 and H3K23 lysines, which leads to the methylation of H3R17 by the arginine methyltransferase CARM1 [22], demonstrating that specific acetyl-lysines can attract chromatin modifying factors as predicted by the histone code model. Importantly, Trim24 (Tripartite motif-containing 24), a co-activator of ERα, recognizes histone tails having concurrent non-methylated H3K4 and acetylated H3K23 residues [43]. Upon estrogen treatment of MCF7 breast cancer cells, Trim24 is recruited along with ERα to estrogen responsive genes that are also became enriched in acetyl-H3K23 [43]. In *Drosophila* S2 cells, dCBP associates with the EcR-B1 receptor isoform upon 20E addition, and acetylates the H3K27 lysine on the 5′ region of the *Sox14* ecdysone induced early gene, a key regulator of dendrite pruning during metamorphosis [44]. Accordingly, dCBP is required for the proper expression of *Sox14* in ddaC neurons during the white pupal stage and its silencing causes pruning defects 18 hours after pupariation [44]. We have shown that dCBP acetylates the H3K23 residue *in vivo*, and that this chromatin mark is enriched on the promoters of the *Eip74EF* and *Eip75B* ecdysone induced genes during the late L3 stage. Although a direct causal relationship between acetylation and gene induction was not demonstrated, the role of dCBP in the regulation of the *Eip74EF* and *Eip75B* genes is supported by the finding that both genes are downregulated in *nej-RNAi* larvae and also by data from the modENCODE project [45] showing extensive binding of dCBP on both gene regions. Previous attempts to detect H3K23 specific acetyltransferase activity of dCBP gave contradicting results: while increased acetylation of H3K23 was not detected in S2 cells expressing transiently transfected dCBP; recombinant dCBP acetylated H3K23 in an *in vitro* acetylation assay [32]. Furthermore, the level of H3K23 acetylation was unchanged in ddaC neurons after RNAi silencing of dCBP [44] suggesting that a HAT other than CBP might also possess this activity.

Our findings along with those of others indicate that CBP dependent histone acetylation at the regulatory region of steroid induced genes might be a conserved chromatin modification in response to the hormone. Furthermore, CARMER, the *Drosophila* homologue of CARM1, associates with the Ecdysone Receptor and is required for ecdysone induced upregulation of apoptotic genes [46], suggesting that it may play a role similar to that of its human counterpart in the regulation of steroid response genes [22]. Together, these results imply the existence of an evolutionarily conserved, multistep chromatin modification mechanism in the transcriptional regulation of nuclear hormone receptor activated genes.

## Materials and Methods

### Drosophila Stocks

Fly strains were maintained and crosses carried out on standard cornmeal – yeast – sucrose Drosophila medium at 25°C unless otherwise noted. The mutant strains *gcn5^E333st^*, *enok^2^* and *nej^3^* were obtained from the Bloomington Drosophila Stock Center (Bloomington, IN, USA). The *nejire* RNAi lines KK105115 and 15319R-2 were obtained from the VDRC (Vienna, Austria) or NIG-FLY (Mishima, Japan) stock centers, respectively. The UAS-dCBP and UAS-dCBP-FLAD transgenic lines were provided by Justin P. Kumar (Indiana University, Bloomington, IN, USA). The *chm^14^* and *mof^2^*mutant lines were provided by Jacques Pradel (Development Biology Institute of Marseille Luminy, Marseille, France) and John Lucchesi (Emory University, Atlanta, GA, USA), respectively.

### Transcript Analysis

To measure the transcriptional activity of ecdysone induced genes during the third larval instar RNA was prepared from synchronized larvae. *w^1118^* eggs were collected on agar – raspberry juice plates for eight hours and then transferred to standard Drosophila medium. Synchronization was carried out three days later by collecting larvae that molted from second to third instar during a four-hour interval. Synchronized L3 larvae were transferred to a new plate from which larvae were collected every four hours until pupariation. Three independent replicates of the synchronized developmental series were collected.

To determine the expression level of the *Eip74EF* and *Eip75B* genes at reduced *nejire* levels, we crossed homozygous *UAS-nejire-RNAi^KK105115^* females with *hs-GAL4/TM6 Tb* males at 18°C and heat-shocked the L3 larvae four times for 1 hour at 37°C. *UAS-nejire-RNAi^KK105115^/hs-GAL4* and *UAS-nejire-RNAi^KK105115^/TM6 Tb* control larvae were collected four hours after the last heat shock based on the *Tb* phenotype.

Larvae were homogenized in Trizol Reagent (Invitrogen, Carlsbad, CA, USA) and RNA was prepared according to the manufacturer’s recommendation. The RNA samples were quantitated by a NanoDrop 1000 spectrophotometer (Thermo Scientific, Waltham, MA, USA), and treated with RNAse free DNAseI (Fermentas, Vilnius, Lithuania) to remove genomic DNA contamination. First strand cDNA was generated from 1 µg RNA samples using Taqman Reverse Transcription Reagent (Applied Biosystems, Foster City, CA, USA) with random hexamer primers. Transcript levels were determined by quantitative real-time PCR (Q-PCR) using gene specific primers with Power SYBR Green PCR Master Mix (Applied Biosystems) in an ABI 7500 Real-Time PCR System (Applied Biosystems). Relative cDNA quantities were calculated by setting Ct values against template calibration curves, and normalizing to the level of the housekeeping *Rp49* ribosomal protein gene. The sequences and chromosomal location of primers are in Table S1.

### Chromatin Immunoprecipitation

Chromatin samples were prepared from w*^1118^* larvae in the middle of the L3 instar (mid-L3), wandering L3 larvae (w-L3) and from late L3 larvae with everted anterior spiracles (spev-L3). Mid-L3 larvae were collected 28–32 hours after the L2/L3 molt as described above, w-L3 larvae were collected based on their wandering behavior, while spev-L3 larvae were collected based on the presence of everted spiracles. Homozygous *gcn5^E333St^* and heterozygous control chromatin was prepared from w-L3 larvae. Chromatin preparation and immunoprecipitation was performed as described previously [47]. In short, chromatin samples were prepared from 1 g larvae in the presence of 10 mM Na-butyrate and protease inhibitors. Chromatin samples were cross-linked with 1% formaldehyde for 10 minutes then fragmented by sonication in a Bioruptor (Diagenode, Denville, NJ, USA). 20 µg chromatin was used for immunoprecipitation after pre-clearing with BSA and salmon sperm DNA blocked Protein A – Sepharose CL-4B beads (Sigma-Aldrich, St. Louis, MO, USA). IPs were done at 4°C overnight with antibodies listed below, then chromatin-antibody complexes were collected with blocked Protein A – Sepharose beads at 4°C for 4 h. The supernatant of the mock control was used as total input chromatin (TIC) control. After washing steps the samples were reverse crosslinked, and the amount of extracted DNA was determined by Q-PCR using Power SYBR Green PCR Master Mix (Applied Biosystems). The sequences and chromosomal location of primers are in Table S1. Samples were quantitated using a TIC standard curve, the amount of DNA specifically precipitated by the given antibody was calculated by deducting the amount of DNA in the mock control. The antibodies used were the following: anti-H3 ab1791 (Abcam, Cambridge, UK), anti-H3K9ac ab4441 (Abcam), anti-H3K23ac ab1768 (Abcam), anti-H4K8ac ab1760 (Abcam), anti-H4K12ac ab1761 (Abcam), anti-H4K16ac AHP417 (Serotec, Kidlington, UK), ab1762 (Abcam) and ab61240 (Abcam).

### Immunoblots

Wandering L3 larvae of appropriate genotypes were selected and homogenized with a pestle in homogenization buffer (50 mM Tris-HCl pH 7.9, 2 mM EDTA, 50 mM NaCl, 0.5 mM DTT, 10 mM Na-butyrate and protease inhibitor (Protease inhibitor cocktail set I, Calbiochem, Darmstadt, Germany)) using 5 µl buffer per larva. The homogenates were mixed with same amount of 2X reducing Laemmli sample loading buffer containing 5% β-mercaptoethanol, boiled for 5 minutes then centrifuged at 13000 RPM for 10 min at 4°C. The supernatant samples were separated on 10% Tricine-SDS-PAGE [48], transferred to Amersham Hybond-ECL membrane (GE Healthcare, Little Chalfont, UK) and incubated with the following primary and secondary antibodies: anti-H3K23ac ab47813 (Abcam), anti-H4 mab31827 (Abcam), goat-anti-mouse IgG-HRP P0447 (Dako, Glostrup, Denmark), goat-anti-rabbit IgG-HRP P0448 (Dako). Chemiluminescent detection was done using Immobilon Western Chemiluminescent HRP substrate (Millipore, Billerica, MA, USA).

### Immunohistochemistry

Immunostaining of *Drosophila* embryos was performed as described previously [49] with modifications. In short, embryos were dechorionated in bleach, rinsed in water and fixed in 1∶1 heptane : PBS-formaldehyde (4%) for 30 minutes. After devitellinization in 1∶1 methanol : heptane for 20 seconds, embryos were treated with methanol and then with PBS - 0.3% Triton X-100. Embryos were incubated with blocking solution (PBS, 5% NGS, 0.3% Triton X-100) for 30 minutes at room temperature, with primary antibody overnight at 4°C, then with secondary antibody for 1 hour at room temperature. The antibodies were diluted in blocking solution. The primary and secondary antibodies used for immunohistochemistry were the following: mouse anti-β-galactosidase G4644 (Sigma-Aldrich), goat anti-mouse IgG (H+L)-FITC 115-095-166 (Jackson Immunoresearch, West Grove, PA, USA), chicken anti-GFP ab13970 (Abcam), donkey anti-chicken IgG (H+L)-FITC 703-095-155 (Jackson Immunoresearch), anti-H3K23ac ab46982 (Abcam), anti-H3K27ac ab4729 (Abcam), donkey anti-rabbit IgG (H+L)-Cy3 711-165-152 (Jackson Immunoresearch).

To determine acetyl-H3K23 levels in *nej* mutant and control embryos, hemizygous *nej^3^/Y* embryos were stained together with *FM7c, P{ftz/lacC}YH1/Y* and *FM7c, P{ftz/lacC}YH1/nej^3^* control siblings, then separated based on anti-β-galactosidase immunostaining. The strength of anti-H3K23ac immunoreactivity was quantitated by calculating the mean pixel intensity of stained embryos using the ImageJ software [50]. To determine acetyl-H3K23 levels upon dCBP overexpression, *engrailed-GAL4 UAS-EGFP/+; UAS-dCBP/+* and *engrailed-GAL4 UAS-EGFP/UAS-dCBP-FLAD* embryos were stained with anti-H3K23ac and anti-GFP antibodies. Micrographs were taken with a Leica SP5 confocal microscope.

## Supporting Information

Figure S1 **dCBP acetylates the H3K27 residue** ***in vivo*** **.** In *engrailed-GAL4 UAS-GFP UAS-dCBP* (A) and *engrailed-GAL4 UAS-GFP UAS-dCBP-FLAD* (B) transgene carrying embryos the expression pattern of the UAS transgenes are visualized by GFP fluorescence. Immunostaining using anti-acetyl-H3K27 specific antibody shows that the level of acetyl-H3K27 is increased in embryos overexpressing *UAS-dCBP* (C), while it is unchanged in embryos overexpressing the *UAS-dCBP-FLAD* enzymatically dead construct (D).
(TIF)

Table S1 **Oligonucleotides used as PCR primers in transcriptional analyzes and quantitation of ChIP assays.** ^a^ Chromosomal location data correspond to BDGP/FlyBaseGenBank assembly Release 5.44. ^b^ The distance of the 5′ end of the oligonucleotide primer relative to the transcriptional start site of the transcript in parentheses.
(DOC)
