# Supplementary figures and images for: Ecdysone Induced Gene Expression Is Associated with Acetylation of Histone H3 Lysine 23 in Drosophila melanogaster
Source: PLoS One. 2012 Jul 10;7(7):e40565. doi: 10.1371/journal.pone.0040565 (PMC3393682; doi:10.1371/journal.pone.0040565)

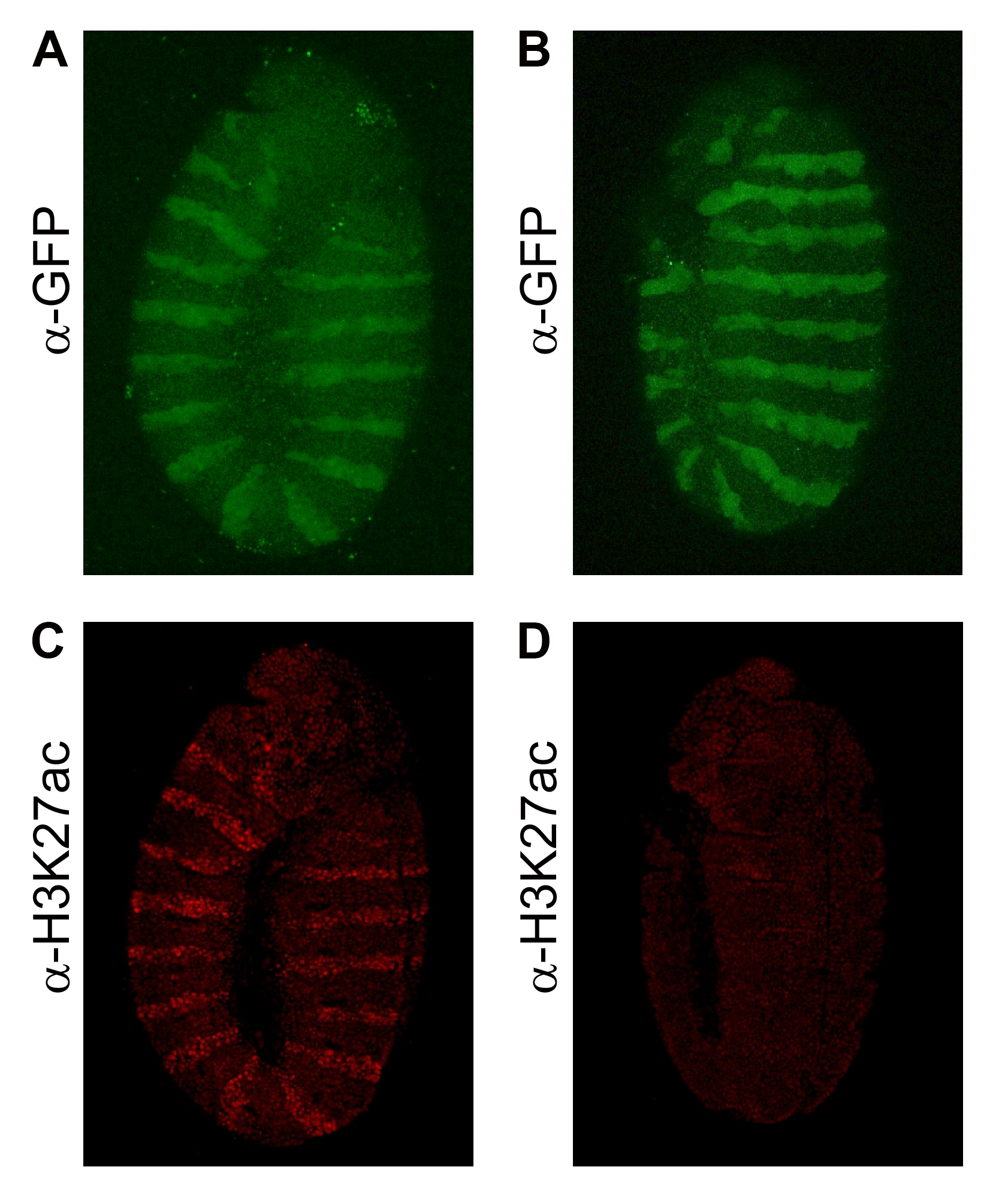

Supplement: Figure S1 — dCBP acetylates the H3K27 residue in vivo . In engrailed-GAL4 UAS-GFP UAS-dCBP (A) and engrailed-GAL4 UAS-GFP UAS-dCBP-FLAD (B) transgene carrying embryos the expression pattern of the UAS transgenes are visualized by GFP fluorescence. Immunostaining using anti-acetyl-H3K27 specific antibody shows that the level of acetyl-H3K27 is increased in embryos overexpressing UAS-dCBP (C), while it is unchanged in embryos overexpressing the UAS-dCBP-FLAD enzymatically dead construct (D). (TIF) [file pone.0040565.s001.tif]
