# Supplementary material for: Ecdysone Induced Gene Expression Is Associated with Acetylation of Histone H3 Lysine 23 in Drosophila melanogaster
Source: PLoS One. 2012 Jul 10;7(7):e40565. doi: 10.1371/journal.pone.0040565 (PMC3393682; doi:10.1371/journal.pone.0040565)
Supplement: Table S1 — Oligonucleotides used as PCR primers in transcriptional analyzes and quantitation of ChIP assays. a Chromosomal location data correspond to BDGP/FlyBaseGenBank assembly Release 5.44. b The distance of the 5′ end of the oligonucleotide primer relative to the transcriptional start site of the transcript in parentheses. (DOC) [file pone.0040565.s002.doc]

| **Primer** | **Primer sequence** | **Chromosomal location**^a^ | **Distance to TSS (bp)**^b^ |
| --- | --- | --- | --- |
| Eip74EF-RA-prom Fw | CAACTAAACGGCGAACAAGC | 3L 17612403..17612422 | +4 (Eip74EF-RA) |
| Eip74EF-RA-prom Rev | GCTTGCGCTCTCTACGCTAA | 3L 17612563..17612544 | -156 (Eip74EF-RA) |
| Eip74EF-int1 Fw | CATACTAGTTGCCGGCGTAT | 3L 17609884..17609865 | +2523 (Eip74EF-RA) |
| Eip74EF-int1 Rev | TTTAGCACTTCCCACTCCTG | 3L 17609701..17609720 | +2706 (Eip74EF-RA) |
| Eip74EF-RB-prom Fw | GCCGACTGAACGACTGAATC | 3L 17569712..17569731 | +14 (Eip74EF-RB) |
| Eip74EF-RB-prom Rev | GGAAGACGCAGCAGAGAGAG | 3L 17569881..17569862 | -155 (Eip74EF-RB) |
| Eip74EF-int5 Fw | CTGGGCTTTGGATACACACT | 3L 17566634..17566615 | +3092 (Eip74EF-RB) |
| Eip74EF-int5 Rev | GTGAGATGCGAGAGAGAAGC | 3L 17566536..17566555 | +3190 (Eip74EF-RB) |
| Eip74EF-ex8 Fw | TGTCCGCGTTTCATCAAGT | 3L 17555115..17555097 | +14611 (Eip74EF-RB) |
| Eip74EF-ex8 Rev | GTTCATGTCCGGCTTGTTCT | 3L 17555005..17555024 | +14712 (Eip74EF-RB) |
| Eip75B-RC-prom Fw | CCGCATAAACGCACAATCAG | 3L 17993000..17993019 | -79 (Eip75B-RC) |
| Eip75B-RC-prom Rev | GTGGATTGGGCTCTCTCTGG | 3L 17993094..17993075 | -173 (Eip75B-RC) |
| Eip75B-RC-int2 Fw | AACCGAGCAAAAACACCAAG | 3L 17990053..17990072 | +2868 (Eip75B-RC) |
| Eip75B-RC-int2 Rev | CGGTTTCCAAGTTCATTGCT | 3L 17990152..17990133 | +2769 (Eip75B-RC) |
| Eip75B-ex8 Fw | CAACTGCACCACCACTTGAC | 3L 17948075..17948056 | +44846 (Eip75B-RC) |
| Eip75B-ex8 Rev | GCCTTGCACTCGTTCTTCTC | 3L 17947972..17947991 | +44949 (Eip75B-RC) |
| Rpl32-prom Fw | TTTCACACCACCAGCTTTTTC | 3R 25871900..25871880 | -38 |
| Rpl32-prom Rev | CACGGACTAACGCAGTTCAA | 3R 25871761..25871780 | +101 |
| Intergen-3L Fw | GCTGATGCTTCCTGAAATCC | 3L 15447688..15447707 | N/A |
| Intergen-3L Rev | GTTTGGTGTGCTCGTCCTTT | 3L 15447809..15447790 | N/A |
| Rp49-DGD Fw | AGCGCACCAAGCACTTCATC | 3R 25871461..25871442 | +401 |
| Rp49-DGD Rev | GACGCACTCTGTTGTCGATACC | 3R 25871304..25871325 | +558 |
